# A Sensor Based on a Spherical Parallel Mechanism for the Measurement of Fluid Velocity: Physical Modelling and Computational Analysis

**DOI:** 10.3390/s18092867

**Published:** 2018-08-30

**Authors:** Roque Saltarén, Gerardo Portilla, Alejandro R. Barroso, Juan Cely

**Affiliations:** 1 Universidad Politécnica de Madrid, Escuela Técnica Superior De Ingenieros Industriales, 28006 Madrid, Spain; 2 Centro de Automática y Robótica, 28006 Madrid, Spain

**Keywords:** mechanical sensor, fluid velocity, parallel mechanism

## Abstract

In this article, a new method was developed to measure the velocity of a fluid using a sensor, based on the use of a spherical parallel mechanism with three degrees-of-freedom (DOF). This sensor transforms the kinetic energy of the fluid into potential energy by deforming the parallel mechanism. This deformation is due to the impact of the fluid on a sphere attached to the platform of the parallel mechanism. Through the acquisition of data from a sensor using an inertial measurement unit (IMU) in the sphere, an algorithm calculates the velocity and direction of the fluid. In this article, a mathematical model of the mechanism and an algorithm for correctly measuring the velocity and direction of the fluid is developed; this algorithm is tested through a simulation in the Adams software, and the MATLAB software is used to execute the algorithm. The results show that the algorithm calculates the velocity and the direction of the fluid correctly, demonstrating the technical feasibility of the sensor.

## 1. Introduction

Fluid velocity sensors can be classified by their measuring principles, whether they use propeller meters, ball meters, electromagnetic meters, or acoustic meters [1]. Unlike these types of sensors, a novel sensor for measuring the velocity and direction of the fluid is proposed in this article. This is possible using a spherical parallel mechanism with three degrees-of-freedom (DOF). The parallel mechanism (PM) can be defined as a multi-degree-of-freedom mechanism, composed of a moving platform and a base connected by at least two serial kinematic chains in parallel [2]. One of the first applications of this type of mechanism is believed to be the tire testing machine introduced by Gough and Whitewall [3], followed by the motion simulation platform built by Stewart [4]. Another example of this class of mechanisms is the Delta robot [5].

A feature of the spherical parallel mechanism is that the degrees-of-freedom of its end effector is rotational. For example, Gosselin and Angeles [6] proposed a spherical three-degree-of-freedom parallel manipulator. This manipulator uses three actuated spherical chains with rotary actuators with axes converging to a point that is the center of rotation. Another parallel mechanism is that of Lande [7]; this has linear actuators ensure a motion of the joint centers, and the rotation around the plate normal is obtained by an articulated stem. Lu and Zhang [8] propose a new parallel mechanism kind: a three-leg, five-DoF 2 SPS + 1 UPU. The researchers Saltaren and Sabater [9] simplified this parallel mechanism to a three-DOF rotational effector with the configuration 2 UPS + 1 RU. We chose to use this last spherical parallel mechanism as a sensor to measure the velocity of a fluid because of its simple mechanical features and its few singularities in the workspace.

There are other devices proposed in the literature that can potentially be used as flow sensors. In these types of devices, the measurement of forces and torques are based on strain gages, for example, such as that implemented in the “compliant-parallel-mechanisms” that appear in [10,11]. The flow measurement for to be implemented in devices, based on stress–strain concepts, should be different from the kinematic models of the parallel mechanisms (as proposed in this article). For this reason, the compliant devices would require specific adaptations and different algorithm formulation in order to measure flows.

Regarding the use of a parallel mechanism as a sensor, the research by Eileen and Ying [12] proposed a novel 3D force sensor based on a parallel mechanism, which displayed good sensitivity and high resolution. Yao and Hou [13] conducted a theoretical and experimental investigation into the isotropy performance of a pre-stressed force sensor based on a Stewart platform. Lu and Wang [14] designed and analyzed a novel force/torque sensor for a hybrid hand with three fingers.

With these properties of parallel mechanisms, the contribution of this article is a novel kind of sensor for measuring the velocity of a fluid based on these mechanisms, as evidenced by the patent number ES2525394B2 (see the section on patents). This sensor measures the velocity and direction of the fluid through a parallel mechanism that has passive actuators (using springs). The platform of the mechanism is connected to a spherical body; within this body, there is an inertial measurement unit (IMU). When the fluid collides with the sphere, deforming the parallel mechanism, it is possible to know the angles of the platform and the acceleration of the sphere using the IMU. These angles and acceleration are input into an algorithm that is developed in this article, and the velocity and direction of the fluid are calculated. This is possible with the model’s mathematics and the dynamics of the parallel mechanism.

### 1.1. Motivation

The development of the proposed sensor has arisen from the need to have a real-time velocity measurement system in order to control the navigation of the underwater robot [15]. Usually, the velocity measurement of the underwater robot is made by acoustic signals and sophisticated algorithms. Kalman filters and particle filters are used for the treatment of these signals [16,17]. Normally, this method uses the combination of signals from an inertial navigation system (INS) and a speed log doppler (DVL) system to provide 3D velocity to the navigation control of the underwater vehicle. Theoretically, the INS/DVL signal combination requires sufficient beam measurements (at least three) to calculate the 3D velocity. However, in cases where the DVL only has limited beam measurements (less than three), the slightly coupled navigation system can no longer function, and only the INS works. Therefore, the navigation error accumulates over time [18]. The proposed sensor, unlike the usual acoustic methods, works on the conversion of kinetic energy into pressure. Due to the features of the velocity sensor, it is possible to believe that it would have a very significant impact for underwater robot uses.

### 1.2. Outline

The rest of this article is organized as follows. In Section 2, we describe the sensor as in the patent, as well as the model’s mathematics, the algorithm for the sensor, and the experimental testing of the algorithm. Section 3 shows the results of the experimentation, and Section 4 presents the discussion about the results obtained. Finally, Section 5 presents the patent for this sensor.

## 2. Materials and Methods

### 2.1. Sensor Description 

**Figure 1:**
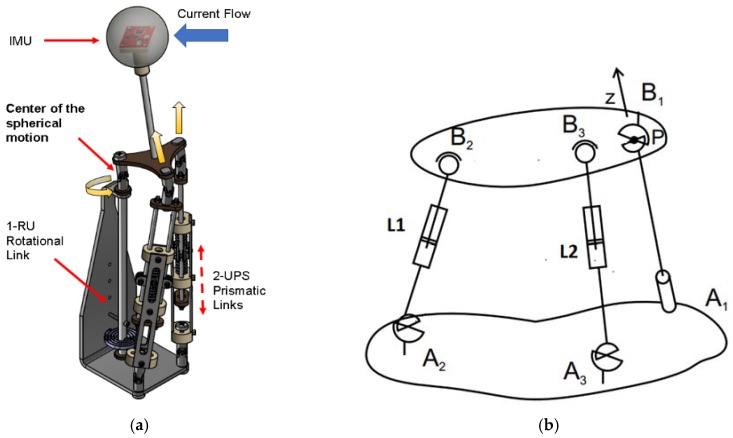
This figure shows the sensor parts and the working method. (**a**) The sensor and its parts; in this figure, we see the sphere, the sensor’s inertial measurement unit (IMU), and the parallel mechanism. (**b**) The parts of the parallel mechanism. *L*_1_ and *L*_2_ are the prismatic passive actuators, and A_1_ is the rotational passive actuator. A_2_, A_3_, and B_1_ are universal joints and B_2_ and B_3_ are spherical joints. Finally point P is the center of the spherical motion.

Figure 1a shows the sensor as described in the patent (see the section on the patent). This sensor has three important parts: the sphere, neck, and the parallel mechanism. The sphere is a hollow part, and inside this there is an inertial measurement unit (IMU). When the fluid drags this sphere, the IMU calculates the inclination angle and the acceleration of the sphere, as seen in Figure 1a. This inclination is transmitted through the neck to the parallel mechanism. This mechanism has three DOF (see Figure 1b) with the configuration 2 UPS + 1 RU. It has three passive actuators, two prismatic (*L*_1_ and *L*_2_) and one rotational (A_1_). These actuators are passive because they only use springs. The joints of the parallel mechanism include three universal joints (B_1_, A_2_, and A_3_): one in the rotational actuator and two in the base of the prismatic actuator. It also has two spherical joints (B_2_ and B_3_) above the prismatic actuators.

The working method of this sensor is as follows. When the fluid drags the sphere, this deforms the parallel mechanism; by using the IMU, it is possible to know the inclination angle of the platform of the parallel mechanism and, by using inverse kinematics, the deformation of the springs is calculated. Knowing the deformation of the springs, we can ascertain the force of each actuator. Then, by using dynamics, the drag force in the sphere is calculated. Finally, the velocity of the fluid by the hydrodynamic equation of the drag force is calculated because it is proportional to the velocity of the fluid.

### 2.2. Kinematics of the Parallel Mechanism 

**Figure 2:**
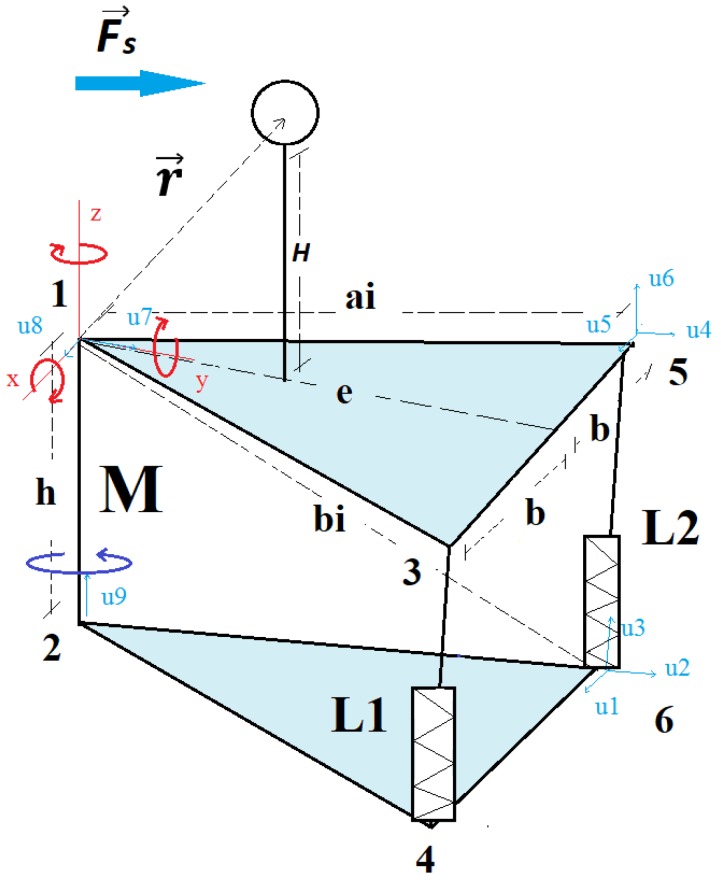
Simplified diagram of the parallel mechanism, where the plane triangles are isosceles and the line of length ‘e’ is the median of the triangle. The sphere is also represented, and within it is the IMU. $\vec{F_{s}}$ is the force resulting from the fluid dynamic forces, and $\vec{r}$ is the vector of the center of the coordinates to the IMU.

Figure 2 shows a simplified diagram of the parallel mechanism, where the axes of the coordinates in Joint 1 can be seen. It should also be noted that the triangle of the platform is isosceles. The line between 1 and 2 represents the rotation joint (*M*), and the lines between 3 and 4 (*L*_1_*)* and between 5 and 6 (*L*_2_) show passive linear actuators.

#### 2.2.1. Calculation of the Inverse Kinematics

To develop the inverse kinematics, the position of each joint is calculated with the rotation matrices [19,20], all in the frame of reference of X, Y, and Z located in Joint 1.

After calculating at each point, we obtain

(1) $$\left[\begin{array}{c} x_{3}\\ y_{3}\\ z_{3} \end{array}\right]=R\left(q_{x},q_{y},q_{z}\right)\left[\begin{array}{c} b\\ e\\ 0 \end{array}\right],\;\left[\begin{array}{c} x_{5}\\ y_{5}\\ z_{5} \end{array}\right]=R\left(q_{x},q_{y},q_{z}\right)\left[\begin{array}{c} -b\\ e\\ 0 \end{array}\right],$$

where *q_x_*, *q_y_*, and *q_z_* are the angles of orientation of the platform in Joint 1. Therefore, the actuator would be determined by the equation

(2) $$L_{1}=\sqrt{{\left(x_{3}-x_{4}\right)}^{2}+{\left(y_{3}-y_{4}\right)}^{2}+{\left(z_{3}-z_{4}\right)}^{2}},$$

(3) $$L_{2}=\sqrt{{\left(x_{5}-x_{6}\right)}^{2}+{\left(y_{5}-y_{6}\right)}^{2}+{\left(z_{5}-z_{6}\right)}^{2}},$$

(4) $$M=q_{z},$$

In this way, the displacements of the actuators *L*_1_ and *L*_2_ and the rotation of *M* are calculated, depending on the orientation angles.

#### 2.2.2. Calculation of the Jacobian Matrix

The calculation of the Jacobian matrix was performed by using the screw theory [21,22]. Therefore, after calculating the twist in each joint, we obtain

(5) $${\hat{\$}}_{1}=\left[\begin{array}{c} u_{1i}\\ b_{i\;}x\;u_{1i} \end{array}\right],\;{\hat{\$}}_{2}=\left[\begin{array}{c} u_{2i}\\ b_{i\;}x\;u_{2i} \end{array}\right],\;{\hat{\$}}_{3}=\left[\begin{array}{c} 0\\ u_{3i} \end{array}\right],\;{\hat{\$}}_{4}=\left[\begin{array}{c} u_{4i}\\ a_{i\;}x\;u_{4i} \end{array}\right],{\hat{\$}}_{5}=\left[\begin{array}{c} u_{5i}\\ a_{i\;}x\;u_{5i} \end{array}\right],{\hat{\$}}_{6}=\left[\begin{array}{c} u_{6i}\\ a_{i\;}x\;u_{6i} \end{array}\right],$$

$${\hat{\$}}_{7}=\left[\begin{array}{c} u_{7}\\ 0 \end{array}\right],\;{\hat{\$}}_{8}=\left[\begin{array}{c} u_{8}\\ 0 \end{array}\right],\;{\hat{\$}}_{9}=\left[\begin{array}{c} u_{9}\\ 0 \end{array}\right],$$

Now, we identify a screw ($*r*) that is reciprocal [23,24] to all the joints, which is given by

(6) $${\hat{\$}}_{r}=\left[\begin{array}{cc} {\left(a_{2}xu_{3,i}\right)}^{T} & u_{3,i}{}^{T}\\ u_{9}{}^{T} & 0^{T} \end{array}\right],$$

After applying the product between reciprocals [25,26], we see that

(7) $${\hat{\$}}_{r,i}^{T}{\;\$}_{p}=v_{i}{\hat{\$}}_{r,i}^{T}{\;\$}_{i},$$

In this way, the Jacobian matrix is obtained

(8) $$\;J=\left[\begin{array}{c} \begin{array}{c} {\left(a_{2}xu_{3,1\;}\right)}^{T} \end{array}\\ \begin{array}{c} {\left(a_{2}xu_{3,2}\right)}^{T}\\ u_{9}{}^{T} \end{array} \end{array}\right],$$

#### 2.2.3. Analysis of Workspace and Singularities of the Parallel Mechanism

An important limitation of the parallel mechanism is that singular configurations may exist within the workspace, where the mechanism can gain one or more degrees-of-freedom and become uncontrollable. Furthermore, the actuator forces may become very large and may result in a breakdown of the mechanism. Therefore, it is of primary importance to avoid singularities in a given workspace [27].

For this reason, we analyze the workspace that is calculated through the inverse kinematic explained above, and the singularity is determined using Equation (9), where the determinant of the Jacobian matrix is zero. At that point, there is a singularity.

**Figure 3:**
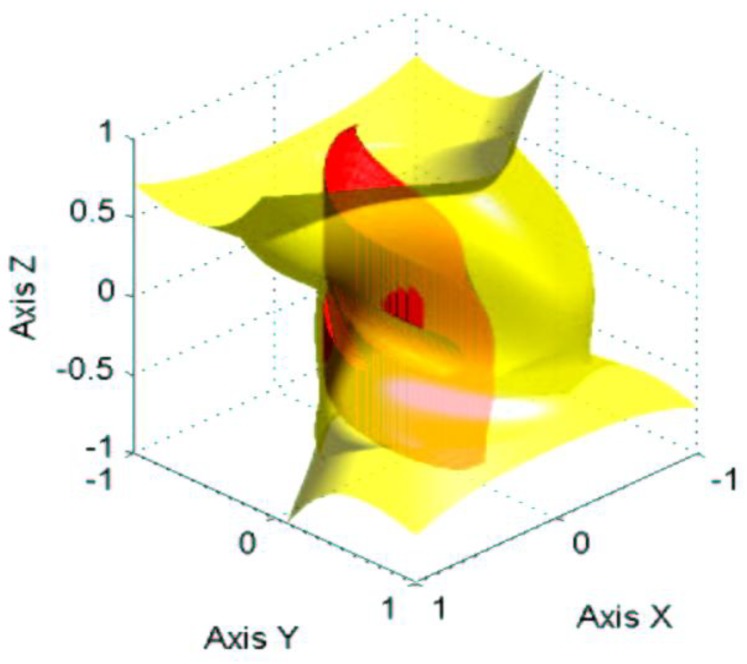
Workspace (red surface) and singularities (yellow surface) of the parallel mechanism. In this figure, almost all the of the singularity surface is placed outside the workspace. There are very few singularities in the possible rotation of the parallel mechanism.

(9)  det(J) =0,

Figure 3 shows the workspace (red surface) and the singularities (yellow surface) of the parallel mechanism. We can see that this mechanism has very few singularities, because the singularities are almost entirely placed outside the workspace.

### 2.3. Dynamics

The dynamics of this parallel mechanism were solved by means of the virtual work principle [28,29] in which the equation that describes the dynamics of a parallel mechanism is the following [30]

(10) $$\;J^{T}\tau +F_{p}+\overset{2}{\underset{i=1\;}{\sum }}\left(J_{i1}^{T}\;F_{i1}+J_{i2}^{T}\;F_{i2}\right)+J_{3}^{T}\;F_{3\;}=\tau_{a}\;,$$

where *J* is the Jacobian matrix of the parallel mechanism, *τ* is a vector of forces and torques of the actuators, $\tau_{a}$ is a vector of torques of Joint 1, $F_{P}$ is the force of platform, $J_{i1}^{T}$ is the Jacobian matrix of the link *i* of Actuator 1, $F_{i1}$ is the actuator cylinder force *i*, $J_{i2}^{T}$ is the Jacobian matrix of the link *i* of Actuator 2, $F_{i2}$ is the actuator piston force *I*, $J_{3}^{T}$ is the Jacobian of the rotational actuator in Joint 2, and $F_{3}$ is the torque of the rotational actuator in Joint 2.

#### 2.3.1. Resulting Force of Each Body Due to Buoyancy

The fluids generate a pushing force. The resultant force between the weight and that force [31,32] would be resolved using

(11) $$\;\vec{f_{m_{i}\;}}=m_{i}\;\vec{g}-\rho \vec{g}\;v_{i}\;,$$

where $\vec{f_{m_{i}}}$ is the resulting force due to buoyancy, $m_{i}$ is the mass of the body *i*, $\vec{g}$ is the gravity vector, ρ is the density of the fluid, and $v_{i}$ is the volume of the body *i.* Clearing mass and gravity, we get

(12) $$\;\vec{f_{mi\;}}=m_{i}\vec{g}\left(1-\left(\frac{\rho \;}{\rho_{i}\;}\right)\right)\;\;,$$

where $\rho_{i}$ is the density of the body *i.* We can see that the relationship of densities is a constant. Hence, to simplify the equation, it can be replaced by a constant $\lambda_{i}$ in such a way that the resulting force would be

(13) $$\;\vec{f_{mi\;}}=m_{i}\vec{g}\;\lambda_{i},$$

#### 2.3.2. Matrix of Forces and Torques of Each Actuator

The force of actuators is calculated with the spring displacement of each actuator.

(14) $$\;\tau =\left|\begin{array}{c} f_{1}\;\\ f_{2}\\ \tau_{m} \end{array}\right|,$$

where $f_{1}$ is the force of actuator *L*_1_, $f_{2}$ is the force of actuator *L*_2_, and $\tau_{m}$ is the torque of actuator *M*.

These forces are calculated as

(15) $$f_{1}=-\Delta L_{1}\;k_{L}-\Delta v_{3,1}\;B_{L},$$

(16) $$\;f_{2}=-\Delta L_{2}\;k_{L}-\Delta v_{3,2\;}B_{L},$$

(17) $$\;f_{2}=-\Delta L_{2}\;k_{L}-\Delta v_{3,2\;}B_{L},$$

where *k* is the stiffness coefficient and *B* is the damping coefficient of the springs.

#### 2.3.3. Platform of the Parallel Mechanism

Gravity and buoyancy are the forces of action, and the degrees-of-freedom of the platform are angular. The wrench will only have torque and not force, so this is resolved by

(18) $$\;F_{p}=\left[\begin{array}{c} F\\ n \end{array}\right]=\left[\begin{array}{c} 0\\ -I_{cp}{\dot{w}}_{p}-w_{px}I_{cp}w_{p}+C\;x\;m_{p}x\;\vec{g}\lambda_{p} \end{array}\right],$$

where *C* is the centroid of the platform, $m_{p}$ is the mass of the platform, $I_{cp}$ the inertia of the platform, $w_{p}$ is the angular velocity of the platform, g is the gravity vector, and $\lambda_{p}$ is the relationship between the densities of the platform and fluid (see Equation (13)).

#### 2.3.4. Cylinder of Linear Actuator *i*

The cylinder is affected by gravity and its linear acceleration.

(19) $$\;F_{i1\;}=\left[\begin{array}{c} F\\ n \end{array}\right]=\left[\begin{array}{c} m_{i1}\left(\vec{g}\lambda_{i1}-a_{i1}\right)\\ -I_{ci1}{\dot{w}}_{i}-w_{ix}I_{ci1}w_{i} \end{array}\right],$$

where $m_{i1}$ is the mass of the cylinder, $a_{i1}$ is the linear acceleration of the cylinder, $w_{i}$ is the angular velocity of the actuator *L_i_* in the lower joint (4 or 6), $I_{ci1}$ is its inertia, and $\lambda_{i1}$ is the relationship between the densities of the cylinder and fluid (see Equation (13)).

#### 2.3.5. Piston of the Linear Actuator *i*

In the same manner, the following is solved by

(20) $$F_{i2}=\left[\begin{array}{c} F\\ n \end{array}\right]=\left[\begin{array}{c} m_{i2}\left(\vec{g}\lambda_{i2}-a_{i2}\right)\\ -I_{ci2}{\dot{w}}_{i}-w_{ix}I_{ci2}w_{i} \end{array}\right],$$

where $m_{i2}$ is the mass of the piston, $a_{i2}$ is the linear acceleration of the piston, $I_{ci2}$ is its inertia, and $\lambda_{i2}$ is the relationship between the densities of the piston and fluid (see Equation (13)).

#### 2.3.6. Rotating Actuator

This joint is only affected by gravity, buoyancy, and angular velocity,

(21) $$F_{3}=\left[\begin{array}{c} F\\ n \end{array}\;\right]=\left[\begin{array}{c} m_{3}\vec{g}\lambda_{3}\\ -I_{c2}{\dot{w}}_{3}-w_{3x}I_{c2}w_{3} \end{array}\right],$$

where $\;m_{3}$ is the mass of the rotation actuator, ${\dot{w}}_{3}$ is its angular acceleration, $w_{3}$ is its angular velocity, $I_{c2}$ is its inertia, and the $\lambda_{i2}$ is the relationship between the density of the piston and fluid (see Equation (13)).

#### 2.3.7. Velocities and Angular Accelerations

The angular velocities and accelerations of the cylinder are calculated, as well as the piston and its linear accelerations. The vectors of the parallel mechanism will be defined first.

The unitary vectors of each actuator are

(22) $$\;\vec{S_{1}\;}=\frac{\left[(p_{3x}-p_{4x}\right)\;,\left(p_{3y}-p_{4y}\right)\;,\;\left(p_{3z}-p_{4z}\right){]}^{T}}{\left|\left[(p_{3x}-p_{4x}\right),\left(p_{3y}-p_{4y}\right)\;,\left(p_{3z}-p_{4z}\right){]}^{T}\right|}\;,$$

(23) $$\vec{S_{2}\;}=\frac{\left[(p_{5x}-p_{6x}\right)\;,\;\left(p_{5y}-p_{6y}\right)\;,\;\;\left(p_{5z}-p_{6z}\right){]}^{T}}{\left|\left[(p_{5x}-p_{6x}\right)\;\;,\;\;\left(p_{5y}-p_{6y}\right)\;,\;\;\left(p_{5z}-p_{6z}\right){]}^{T}\right|},$$

(24) $$\;\vec{S_{3}\;}={\left[0,0,1\right]}^{T}\;,$$

where $\vec{S_{1}}$ is the unit vector of the actuator *L*_1_, $\vec{S_{2}}$ of the unit vector actuator *L*_2_, and $\vec{S_{3}}$ is the unit vector of actuator *M* in the center of the frame of reference in Joint 1.

Then, the vectors of the platform are

(25) $$\vec{a_{1}}=\left[(p_{3x}-p_{1x}\right)\;,\left(p_{3y}-p_{1y}\right)\;,\;\left(p_{3z}-p_{1z}\right)],$$

(26) $$\;\vec{a_{2}\;}=\left[(p_{5x}-p_{1x}\right)\;,\left(p_{5y}-p_{1y}\right)\;,\;\left(p_{5z}-p_{1z}\right)]\;,$$

(27) $$\vec{h}=\left[(p_{2x}-p_{1x}\right)\;,\left(p_{2y}-p_{1y}\right)\;,\;\left(p_{2z}-p_{1z}\right)],$$

Now, the angular speeds of the actuators are calculated.

In the case of actuator *L*_1_, the tangential speed [33] of actuator *L*_1_ and that of the platform in Joint 3 are equalized. Therefore, we have Equation (28):

(28) $$w_{1}x\;\vec{L_{1}\;}=w_{p}\;x\;\vec{a_{1}}\;,$$

Solving this, we get

(29) $$\;w_{1}x\;L_{1}x\;\vec{S_{1}\;}=w_{p}\;x\;\vec{a_{1}}\;,$$

(30) $$\;w_{1}=-\left(\frac{1}{L_{1}\;}\right)\;\vec{S_{1}}x\;w_{p}\;x\;\vec{a_{1}}\;,$$

Thus, Equation (30) describes the angular velocity of actuator *L*_1_ in Joint 4. In the same manner, actuator *L*_2_ is solved and obtained

(31) $$\;w_{2}=-\left(\frac{1}{L_{2}\;}\right)\;\vec{S_{2}}x\;w_{p}\;x\;\;\vec{a_{2}}\;,$$

The angular velocity of the motor would be given simply by the following equation.

(32) $$\;w_{3}=\vec{S_{3}\;}x\;w_{p}\;,\;$$

Next, the angular accelerations of each actuator in its lower joints (4 or 6) will be calculated in the same way as Equation (28). However, matching the tangential accelerations, we see that the angular acceleration in actuator *L*_1_ would be

(33) $$\;\dot{w_{1}\;}=\left(\frac{1}{L_{1}}\right)x\;{\vec{S}}_{1}x\;ac_{3}-2\dot{\;L1}w_{1}\;,$$

where $ac_{3}$ is the acceleration in Joint 3 which is determined by

(34) $$\;ac_{3}={\dot{w}}_{p}\;x\;\vec{a_{1}\;}+w_{p}x\left(w_{p}x\vec{a_{1}}\right),$$

In the same way, the acceleration is solved for actuator *L*_2_ as

(35) $$\;\dot{w_{2}\;}=\left(\frac{1}{L_{2}}\right)x\;{\vec{S}}_{2}x\;ac_{5}-2\dot{\;L1}w_{2}\;,$$

where $ac_{3}$ is the acceleration in Joint 3 which is determined by

(36) $$\;ac_{5}={\dot{w}}_{p}\;x\;\vec{a_{2}\;}+w_{p}x\left(w_{p}x\vec{a_{2}}\right)\;,$$

and the angular acceleration of the motor is only determined by

(37) $$\;\dot{w_{3}\;}=\frac{dw_{3}}{dt}\;\;,$$

#### 2.3.8. Jacobian Matrix of Each Actuator

The velocity of the center of mass of the cylinder would be given by

(38) $$\;v_{i1\;}=c_{i1}\left(w_{i}\;x\;\vec{S_{i}}\right)\;,$$

(39) $$\;v_{i1\;}=-\frac{C_{i}}{L_{i}}\left[{\vec{S_{i}}}^{2}-{\vec{S_{i}}}^{2}a_{i}\right]\dot{X},$$

where $C_{i}$ is the center of mass of the cylinder and $\dot{X}$ is the velocity of the final effector. Now, we solve for the piston using

(40) $$\;v_{i2\;}=c_{i2}\left(w_{i}\;x\;\vec{S_{i}}\right)+\dot{Li}\vec{S_{i}}\;,\;$$

(41) $$\;v_{i2\;}=\left[-\frac{c_{i2}}{Li}{\vec{S_{i}}}^{2}\;+\vec{S_{i}}\;\vec{S_{i}}{\;}^{T}\;\frac{c_{i2}}{Li}\;x\;{\vec{S_{i}}}^{2}x\;a_{i}\;-\vec{S_{i}}\;\vec{S_{i}}{\;}^{T}\;x\;a_{i}\;\right],$$

Finally, the angular velocity of the actuators with respect to that of the effector would be given by

(42) $$\;w_{i}=\frac{1}{L_{i}\;}\left(\vec{S_{i}\;}x\;Vp+\vec{S_{i}}x\left(wpx\;a_{i}\right)\right),$$

(43) $$\;w_{i}=\frac{1}{L_{i}\;}\left[\vec{\;\;S_{i}\;}-\vec{\;\;S_{i}}x\;\;a_{i}\;\right]\;\left[\begin{array}{c} Vp\\ Wp \end{array}\right]\;,$$

where Vp is the speed of the end effector of the parallel mechanism. In this case, as it is angular, this speed is zero, and Wp is the angular velocity of the end effector. Therefore, the Jacobian matrix of the cylinder is

(44) $$\;J_{i1\;}=\frac{1}{L_{i}}\;\left[\begin{array}{cc} -C_{i}{\vec{S_{i}}}^{2} & C_{i}{\vec{S_{i}}}^{2}a_{i}\\ \vec{S_{i}\;} & -\vec{S_{i}}x\;a_{i} \end{array}\right],$$

and the Jacobian matrix of the piston is

(45) $$\;J_{i2\;}=\frac{1}{L_{i}}\;\left[\begin{array}{cc} -{\vec{c_{i2}S_{i}}}^{2}\;+\vec{S_{i}}\;\vec{S_{i}}{\;}^{T} & \;c_{i2}x\;{\vec{S_{i}}}^{2}x\;a_{i}-\vec{S_{i}}\;\vec{S_{i}}{\;}^{T}\;x\;a_{i}\\ \vec{S_{i}\;} & -\vec{S_{i}}x\;a_{i} \end{array}\right],$$

and the Jacobian axis of the rotational actuator, as it is directly attached to the effector of the parallel mechanism, would be

(46) $$\;J_{3}=\left[\vec{S_{3}\;}\right]\;,$$

#### 2.3.9. Torque Generated by Fluid Dynamic Forces in the Sphere

The fluid dynamic forces in a body are calculated as follows [34]

(47) $$\;M\;\dot{v}+C(v)\;v+D\left(v\right)v+G=F_{s},$$

where $F_{s}$ is the force resulting from the fluid dynamic forces in a body, *M* is the is the inertia matrix (including added mass), *C* is the vector of the Coriolis and centripetal force, D is the damping effect, and *G* is the buoyancy effect.

As this case is a sphere, the equation is resolved as

(48) $$\left(m_{s}+m_{s}\prime \;\right)\vec{a_{s}}+{\vec{f}}_{a}+m_{s}\vec{g}\lambda_{s}=\vec{F_{S}},$$

where $m_{s}$ is the mass of the sphere, $m_{s}\prime$ is the added mass in the sphere by the fluid, $\vec{a_{s}}$ is the acceleration vector of the sphere obtained by the IMU, ${\vec{f}}_{a}$ is the vector of the drag force of the fluid in the sphere, and $\lambda_{s}$ is the relationship of the densities between the sphere and fluid (see Equation (13)).

In this case, the force is not considered to be Coriolis because it is assumed that the sensor is not in an external referential system that rotates, and the centripetal force is very small with respect to the drag force. This is because the distance of the axis of rotation of the parallel mechanism and the sphere is small. This allows us to obtain the drag force as the only dependent factor on the velocity of the fluid.

The added mass in the sphere by the fluid can be calculated as [35]

(49) $$\;m_{s}^{\prime }=0.5\rho v_{s\;\;},$$

where ρ is the density of the fluid and $v_{s}$ is the volume of the sphere.

Finally, the torque generated in Joint 1 (see Figure 2) of the parallel mechanism in the frame of reference is determined by

(50) $$\;\tau_{a}=\vec{r}\;x\;\vec{F_{S}\;}\;,\;$$

where $\vec{r}$ is the vector of the center of the frame of reference to the sensor’s IMU (see Figure 2).

Since we are interested in finding the drag force, it will be necessary to invert $\vec{r}$. For this reason, we arrange the equation as an antisymmetric matrix [36]. Finally, replacing Equation (10), the drag force is calculated by

(51) $$\;{[r]\;}^{-1}{}_{x\;}\;(\;J^{T}\tau +F_{p}+\overset{2}{\underset{i=1}{\sum }}\left(J_{i1}^{T}\;F_{i1}+J_{i2}^{T}\;F_{i2}\right)+J_{3}^{T}\;F_{2\;})-m_{s}\vec{g}\lambda_{s}-\left(m_{s}+{m^{\prime }}_{s}\right)\;\vec{a_{s}}=\vec{f_{a}}\;,$$

#### 2.3.10. Velocity and Direction of the Fluid

The velocity and direction of the fluid is possible to calculate with the equation of the drag force of the fluids [34]

(52) $$\;v=\sqrt{\frac{\|f_{a}\|\;}{C_{D}0.5\rho A}},$$

where $C_{D}$ is the drag coefficient of the sphere, ρ is the fluid density, and A is the cross area of the sphere.

**Figure 4:**
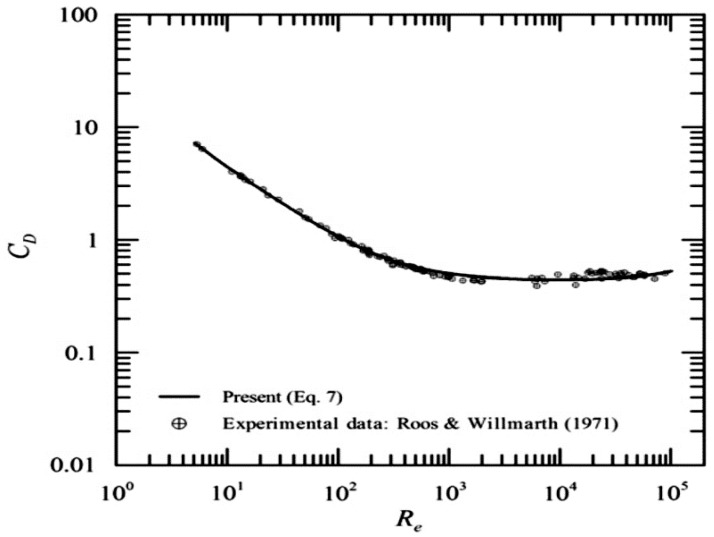
Drag coefficient (*C_D_*) of a sphere versus the Reynolds number (*R_e_*). We can see that the drag coefficient is constant, starting from 10^3^ *R_e_*; with an approximate value of 0.47 [37].

The drag coefficient of the sphere varies with the Reynolds number, as can be seen in Figure 4. This coefficient is constant when the Reynolds number is 10^3^ *R_e_*, with a value of 0.47. The researchers Milkailov and Silvia Freire [37] show different equations for calculating the drag coefficient. According to this criterion, the sphere is designed to keep the drag coefficient constant.

Finally, the direction of the fluid is calculated with the unit vector of the drag force ($f_{a}$)

(53) $$\;\hat{u}=\frac{{\vec{f}}_{a}\;}{\left\|f_{a}\right\|}\;,$$

#### 2.3.11. Dynamic Performance

Since the sensor is a parallel mechanism and has mass and springs, this will have a dynamic behavior of the second order, in such a way that the transference function of the second-order system is as [38]

(54) $$\;Y(s)\;=\frac{k_{p}\;w_{n}{}^{2}}{s^{2}+2\zeta w_{n}s+w_{n}{}^{2}}\;,$$

where ζ is the damping ratio, $w_{n}$ is the natural frequency of the system, and $k_{p}$ is the gain. For good design criteria ζ is set to 0.7 because, in this condition, the system is more robust to the variations in the parameters of the plant or actuator. Thus, with this value, it is possible to calculate the damping of the parallel mechanism with the following equation [38]

(55) $$\;B=\zeta \;2\;\sqrt{Km\;}\;,$$

where *B* is the damping of the parallel mechanism, *K* is the stiffness of the parallel mechanism, and m is the mass of the mechanism. The relationship of the stiffness of the parallel mechanism with the stiffness (*k_i_*) of the actuators is related to the Jacobian matrix, as [27]

(56) $$\;K=J^{T}k_{i}\;J\;,\;$$

where $k_{i}={\left[k_{L},k_{L},k_{m}\right]}^{T}$.

The stiffness of the parallel mechanism is determined by the response time we want for the sensor. This is the settling time ($t_{s}$) and, for a second-order system, the response is usually 2% and can be obtained using the equation [38]

(57) $$\;t_{s}=\frac{4}{\zeta w_{n}},$$

where [39]

(58) $$\;w_{n\;}=\sqrt{\frac{K}{I}}\;,$$

*I* is the rotational mass moment of inertia since the parallel mechanism has rotational grades of freedom. Hence, depending on the settling time that we want the sensor to work with, we will determine the natural frequency ($w_{n}$) of the parallel mechanism and, with Equation (58), determine the stiffness of the parallel mechanism. Finally, with Equation (56), we calculate the stiffness (*k_i_*) of each actuator.

### 2.4. Practical Implementation and Testing of the Sensor

#### 2.4.1. Algorithm for the Sensor

**Figure 5:**
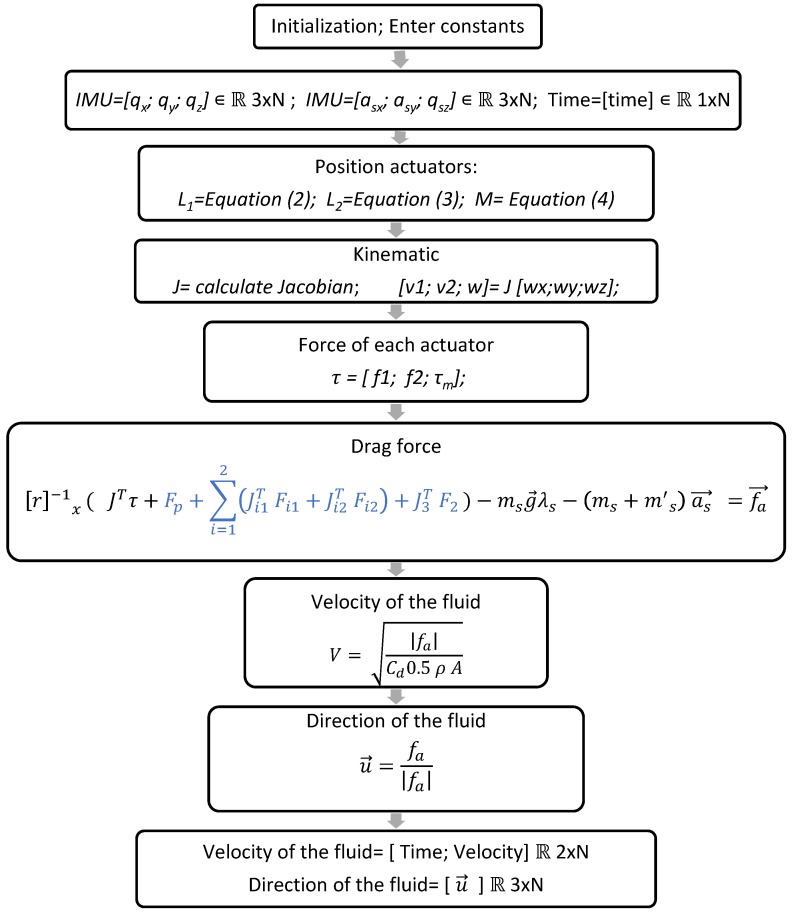
Algorithm for calculating the velocity and direction of the fluid with the parallel mechanism.

Figure 5 shows the algorithm for calculating the velocity and direction of a fluid through a parallel mechanism, using the model mathematics described in this article. The algorithm begins by entering the constants, such as inertias, masses, spring proprieties, geometry, etc. Next, the values of the angles and acceleration measured by the IMU are obtained. Then, we calculate the displacement of the spring of each passive actuator with the equation of the inverse kinematic (see Equations (2)–(4)). We also calculate the Jacobian matrix (see Equation (8)) and, with the Jacobian matrix, we calculate the velocity of each actuator. Finally, with this data, it is possible to calculate the force and torque of the actuators (see Equation (14)).

Next, the algorithm calculates the drag force with the equation dynamic (see Equation (51)). Note that a part of the equation has been shown in blue; this part represents the dynamic forces due to the inertia of the parallel mechanism and, if the inertia of the mechanism is very low with respect to the drag force, it is possible not to consider this part of the equation because the forces that will generate the inertia of the mechanism will be very low. However, this should be done only if it is necessary to reduce the computational cost.

After obtaining the vector of forces of the drag force, the velocity is calculated with the hydrodynamic equation (see Equation (52)) and the direction of the fluid (see Equation (53)). Finally, with this result, we obtain the velocity and the direction of the fluid only with the acquisition of data on the angles and accelerations of the parallel mechanism through the IMU sensor.

#### 2.4.2. Experiment

**Figure 6:**
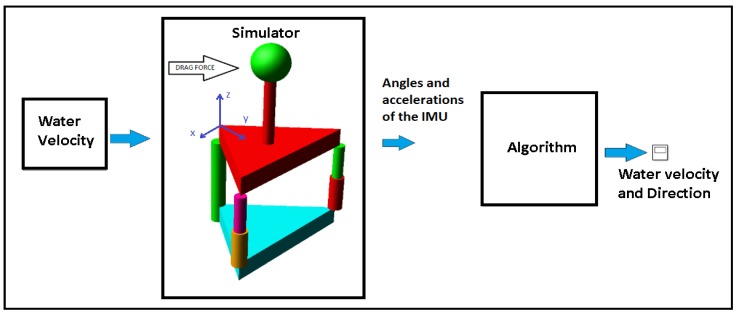
This figure shows the method of simulation for testing the algorithm. The sensor is designed in the Adams software, where all the effects of the dynamics and hydrodynamics of the mechanism are simulated. In this Adams model, the water velocity is input, and the model in Adams software transforms this velocity into drag forces applied in the sphere. The model has a sensor in the sphere that calculates the angles and accelerations as an IMU sensor. These angles and accelerations are input into the algorithm, and the velocity and direction of the water are calculated.

Testing the algorithm employed a co-simulation of MATLAB and Adams software. The algorithm was written in MATLAB, and the sensor of the parallel mechanism was designed in Adams software. The fluid considered in this simulation is water with a density $\rho =1000\;\mathrm{kg}/m^{3}$. Figure 6 shows the simulation process. Firstly, the velocity of the water is input. In this case, a velocity of 1 m/s in the *x*-axis was input. With sinusoidal variation in the model, Adams transformed this velocity into the drag force applied in the sphere.

This force deforms the structure of the parallel mechanism, and a virtual inertial sensor within the sphere measures the angles and accelerations, simulating the IMU sensor. These angles and accelerations were input into the algorithm in MATLAB, and this calculated the velocity and direction of the water. These results were compared with the velocity of the input. In this experiment, a test was also performed to understand the dynamic behavior of the sensor in terms of frequency response and measuring range.

Table 1 shows the physical characteristics of this sensor for the test. The springs’ constants have been calculated for a settling time of 0.15 s of the response of the sensor, as explained in the section on dynamic performance.

## 3. Results

**Figure 7:**
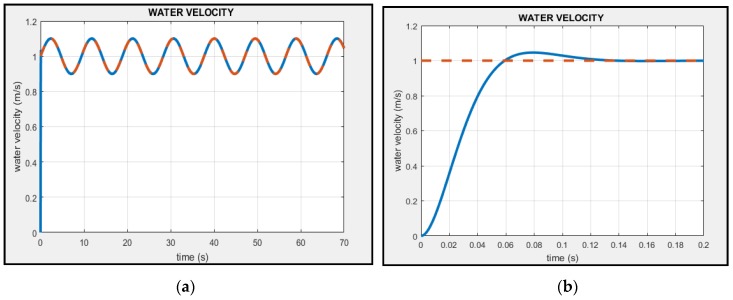
The results of the algorithm. The red line is the input velocity of the water and the blue line is the velocity of the water calculated with the algorithm. (**a**) The results for 70 s. In the first seconds, the sensor measures a little late; it can be seen in (**b**) that the settling time is 0.15 s. Then, as seen in (a), the sensor calculates the velocity correctly.

**Figure 8:**
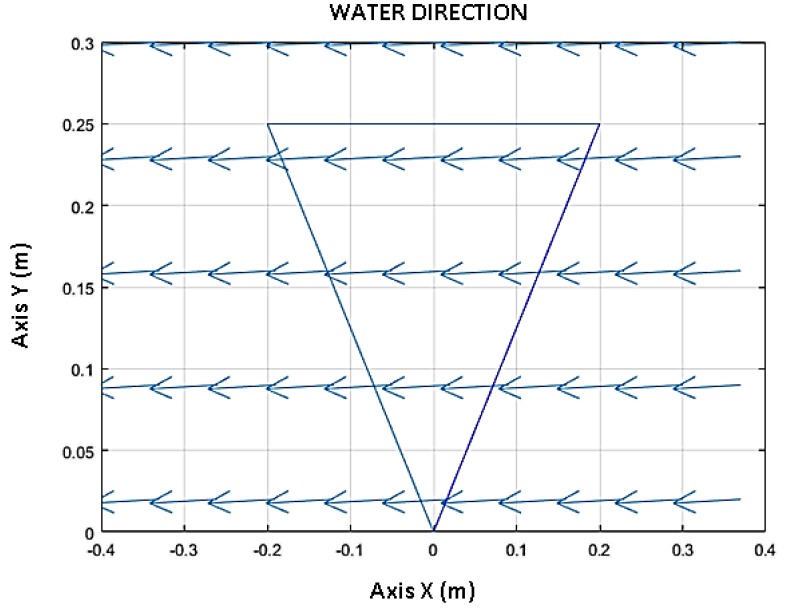
The results of the direction of the water calculated by the algorithm. This vector matches the water velocity vector. The triangle represents the parallel mechanism.

**Figure 9:**
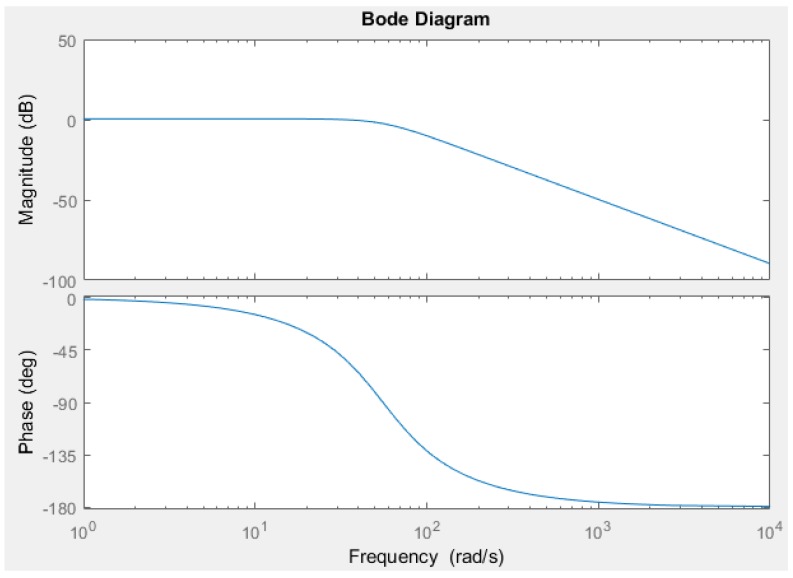
This figure shows the response dynamics of the sensor in terms of frequency. We can see that the sensor can measure correctly up to a lower frequency of 40.6 rad/s. Up to a frequency of 55.6 rad/s the system enters into resonance, as this value is the natural frequency of the parallel mechanism.

**Figure 10:**
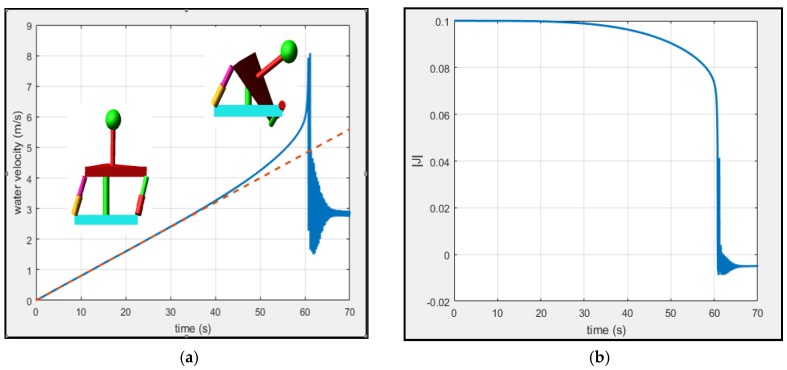
(**a**) The measure range of the sensor. The red line is the input velocity of the water. In this case, the velocity is increasing in a linear form. The blue line is the result of the algorithm. We can see that the sensor response is up to 3.41 m/s. (**b**) The determinant of the Jacobian matrix (see Equation (8)). We can see that thr is zero at 61 s. This means that the parallel mechanism enters into a singularity, failing the sensor, as shown in (a), at 61 s.

The simulation time was 70 s. Figure 7 shows a comparison between the input velocity of the water (red line) and the velocity of the water calculated with the algorithm (blue line). This figure shows the results for 70 s (Figure 7a) and in the first 0.2 s (Figure 7b). Figure 8 shows the direction of the water calculated by the algorithm. The triangle is a reference to the geometry of the sensor. Figure 9 shows the response of the sensor to the frequency to determine the bandwidth of this sensor. Finally, Figure 10 shows the response of the sensor to several velocities. In this case, the velocity of the water that increases in linear form (red line) was input. The blue line represents the velocity of the water calculated by the algorithm. The determinant of the Jacobian matrix is shown in Figure 9b to better understand the singularity of the parallel mechanism.

## 4. Discussion

The results of Figure 7 show that the algorithm can calculate the velocity of the fluid through the angles and accelerations obtained by the sensor’s IMU. The settling time of the sensor is 0.15 s (Figure 7b). This value can increase or decrease, depending of the uses of the sensor. The stiffness of the parallel mechanism can be designed in such a way that the natural frequency of the mechanism increases or decreases the settling time (see Equation (57)).

On the other hand, Figure 8 shows the direction of water calculated by the algorithm. This is calculated to match the direction of the water that was input, as has been previously explained. The water has a direction in the *x*-axis. This is possible to calculate because the drag force has the same direction as the water, so the unitary vector of the drag force and unitary vector of the velocity water are equal.

Figure 9 shows the response of the frequency of the sensor. In this figure, we can see that the sensor response is good until a frequency of 40.6 rad/s. Above this value, the response of the sensor increases and the phase change to 180° degrees. This is because the parallel mechanism comes into resonance. Since the natural frequency of this mechanism is 55.6 rad/s, it is possible calculate the natural frequency with Equation (58). To graph the response of the frequency of the sensor, the transfer function was determined as

(59) $$\;Y(s)\;=\frac{1.0592\;{\left(55.6\right)}^{2}}{s^{2}+2\left(0.7\right)\left(55.6\right)s+{\left(55.6\right)}^{2}}\;,$$

The range of measurement of the sensor can be seen in Figure 10a. Due to inertia and the stiffness of the parallel mechanism, the sensor measures correctly until a velocity of 3.41 m/s. This is because, at higher velocities, the drag force increases until it destroys the sensor. As can be seen in Figure 10b, the determinant of the Jacobian matrix (see Equation (8)) is zero at 61 s. This means that the parallel mechanism entered into a singularity, failing the sensor, as shown in Figure 10a, at 61 s. It is possible to increase this breaking point if we increase the stiffness of the parallel mechanism and also increase the natural frequency, hence decreasing the settling time. The main idea is to maintain the mechanism in its workspace because, as we have seen in Figure 3, there are no singularities that have an effect within the workspace.

## 5. Patents

Gonzalo Emmanuel Ejarque Rinaldini, Roque Jacinto Saltaren Pazmiño, Gabriel Armando Poletti Ruiz, and Rafael Aracil Santonja. “Dispositivo y método para la medición de corrientes de fluidos mediante mecanismo paralelo esférico actuado por fuerzas de arrastre.” ES. Patent no. ES2525394B2. 22 December 2014. 

## Figures and Tables

**Table 1:** Physical characteristics of the sensor flow.

Geometry (m)	Masses (kg)	Spring Constants
*L*_1_ = 0.250 *L*_2_ = 0.250 *h* = 0.25 *h* = 0.250 *b* = 0.20 *a* = 0.40 Diameter of the sphere = 0.1 m	Triangular platforms = 0.1 *L*_1_ = 0.2 *L*_2_ = 0.2 *h* = 0.01 *h* = 0.01 Sphere = 0.001	$k_{L}$ = 320 N/m $B_{L}$ = 8 (N.s)/m $k_{m}$ = 18.33 (N.m)/° $B_{m}$ = 0.458 (N.m.s)/°
